# Supplementary material for: A Long Polymorphic GT Microsatellite within a Gene Promoter Mediates Non-Imprinted Allele-Specific DNA Methylation of a CpG Island in a Goldfish Inter-Strain Hybrid
Source: Int J Mol Sci. 2019 Aug 12;20(16):3923. doi: 10.3390/ijms20163923 (PMC6721770; doi:10.3390/ijms20163923)
Supplement: Supplementary file 1 [file ijms-20-03923-s001.pdf]

Supplementary Data

A Long Polymorphic GT Microsatellite within a Gene Promoter Mediates Non-Imprinted Allele-Specific DNA Methylation of a CpG Island in a Goldfish Inter-Strain Hybrid

Jianbo Zheng [1,2,†,*], Haomang Xu [2,†] and Huiwen Cao [2]

1 Zhejiang Institute of Freshwater Fisheries, Huzhou 313001, China
2 College of Life Sciences, Zhejiang University, Hangzhou 310058, China
* Correspondence: 21207054@zju.edu.cn; Tel.: +86-572-204-5681
† These authors equally contributed to this work.

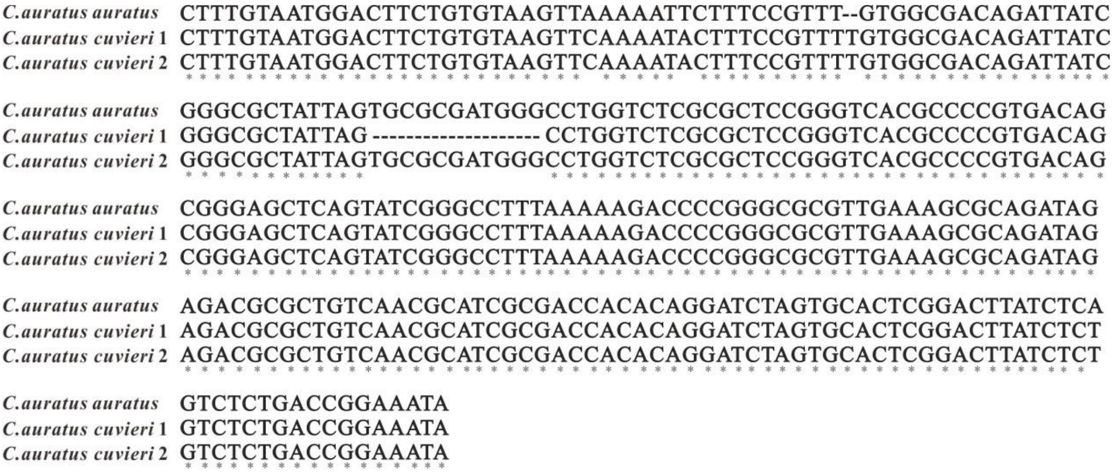

Figure S1. Sequence alignment of *ntl* promoter CpG islands in Chinese GF (*C. auratus auratus*) and Japanese GF (*C. auratus cuvieri*) strains.

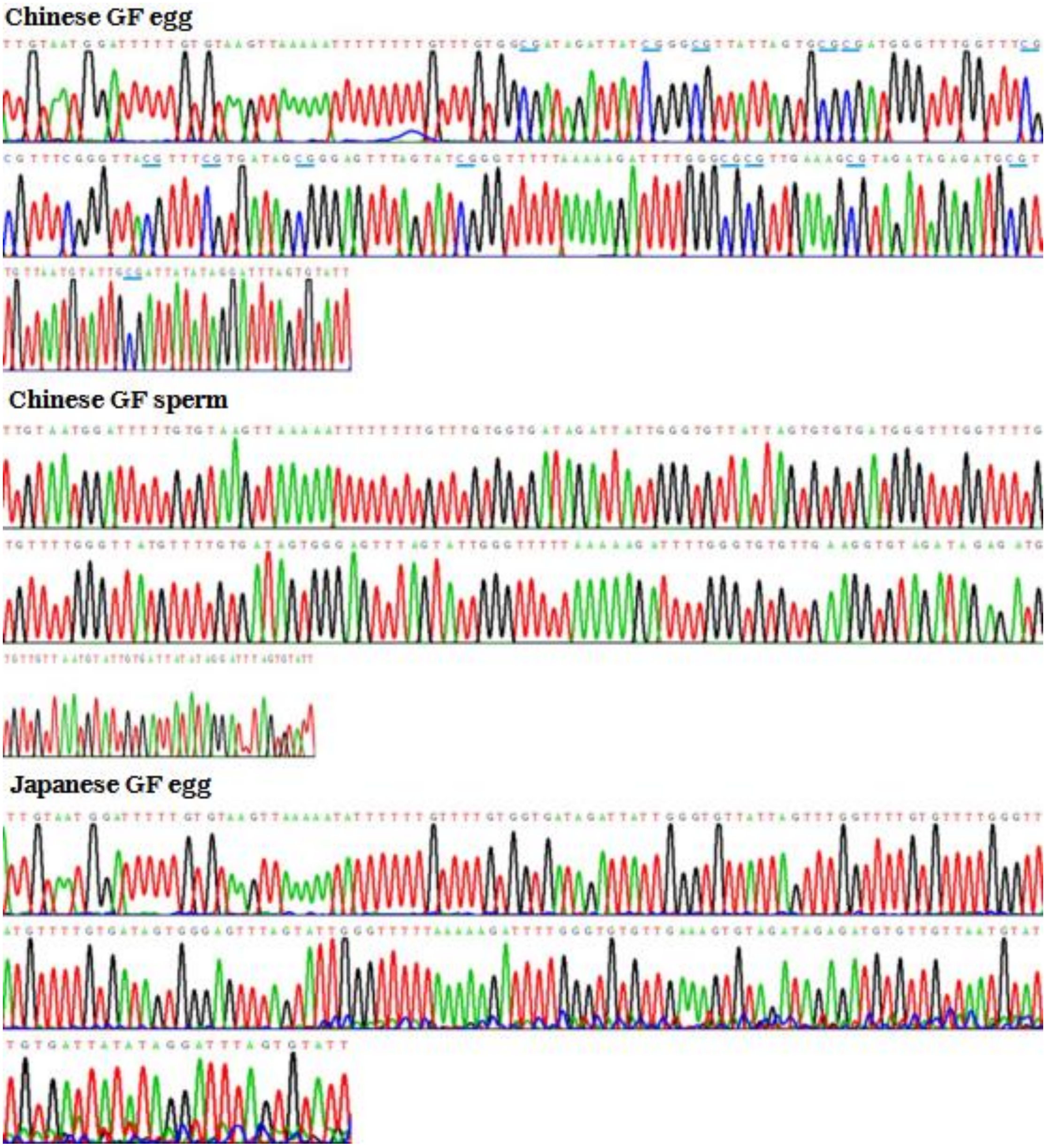

Figure S2. Sequencing spectrums of bisulfate converted goldfish *ntl* promoter CpG-islands in the gametes of a Chinese GF (*C. auratus auratus*) individual and a homozygous Japanese GF (*C. auratus cuvieri*) individual. All the C nucleotides except methylated CpG dinucleotides were converted into T nucleotides after bisulfate treatment. Underscores indicate the methylated CpG dinucleotides.

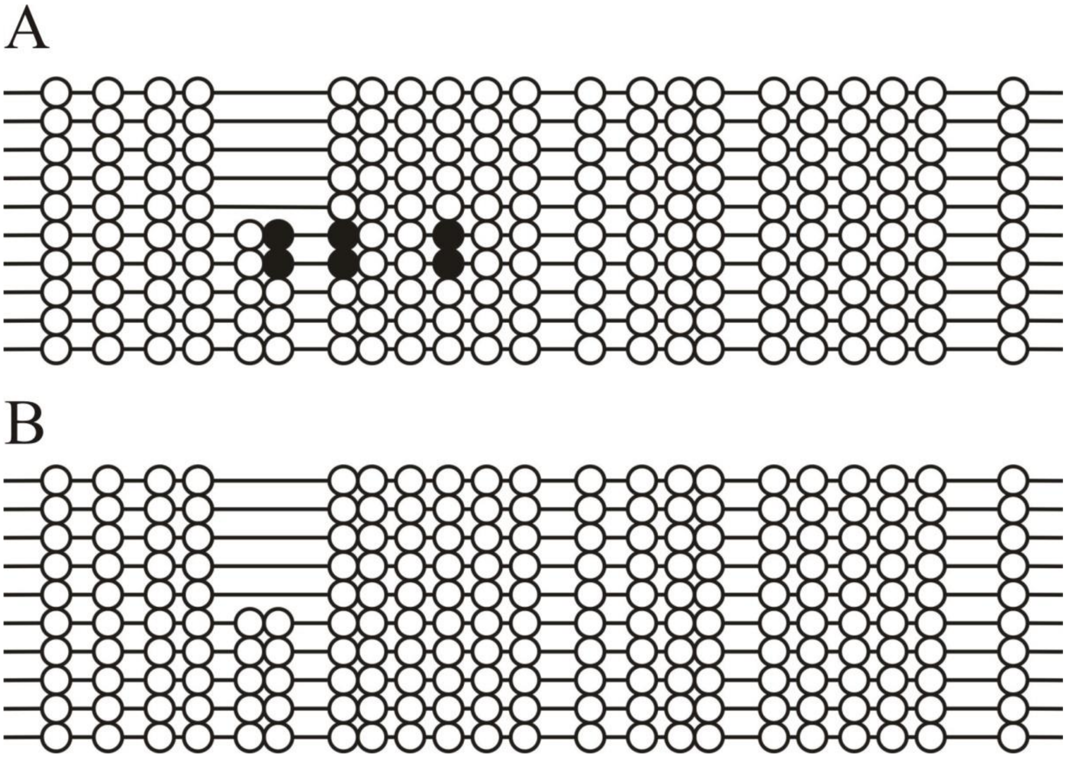

Figure S3. Bisulfite sequencing results of *ntl* promoter CpG-island in the eggs (A) and sperm (B) of heterozygous Japanese GF individuals.

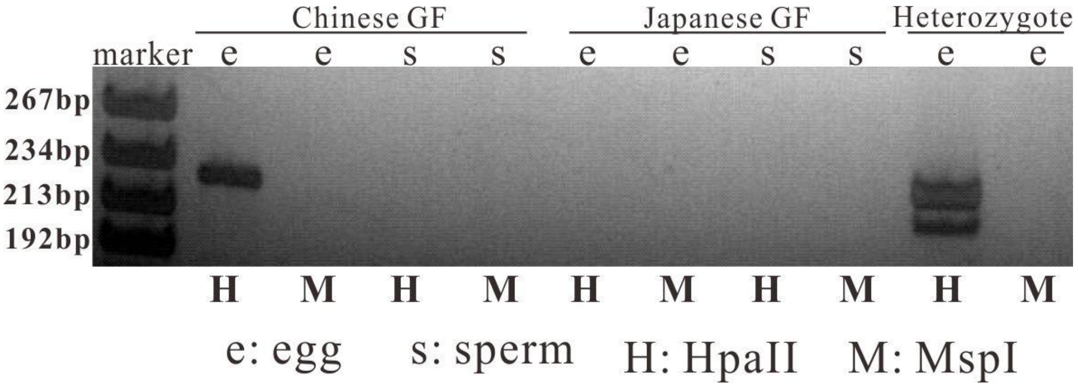

Figure S4. Methylation status of *ntl* promoter CpG island in the gametes of a Chinese GF (*C. auratus auratus*) individual, a Japanese GF (*C. auratus cuvieri*) individual and a heterozygous individual detected by methylation-sensitive PCR.

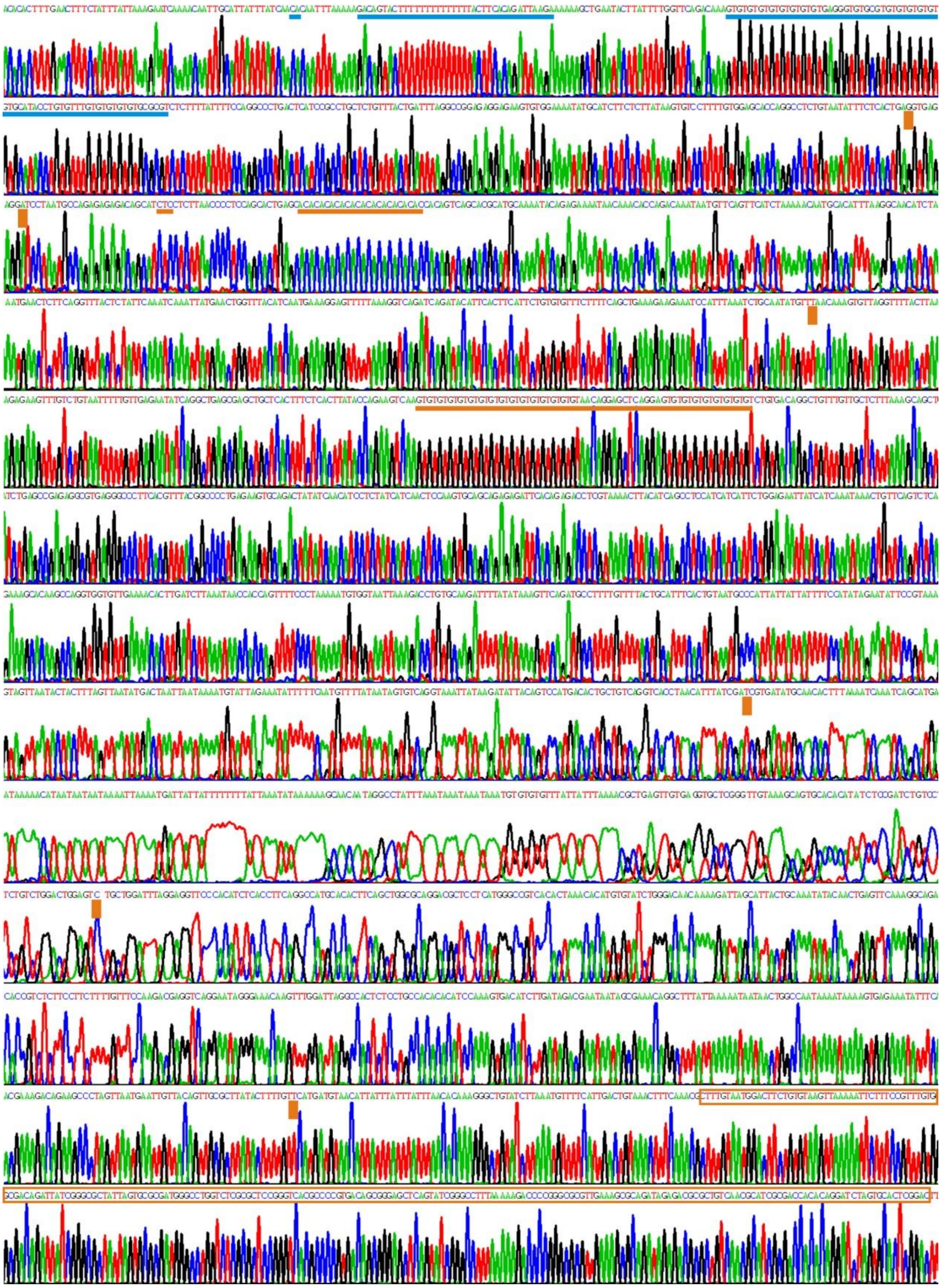

Figure S5. Sequencing spectrums of the recombinant Chinese GF *ntl* promoter in the gynogenetic heterozygous diploid individual 1 (GHD 1). Orange and blue underlines indicate the Chinese GF and Japanese GF specific sequences, respectively. Orange and blue boxes indicate the Chinese GF and Japanese GF specific SNP sites, respectively. Open orange rectangle indicates the Chinese GF CpG island.

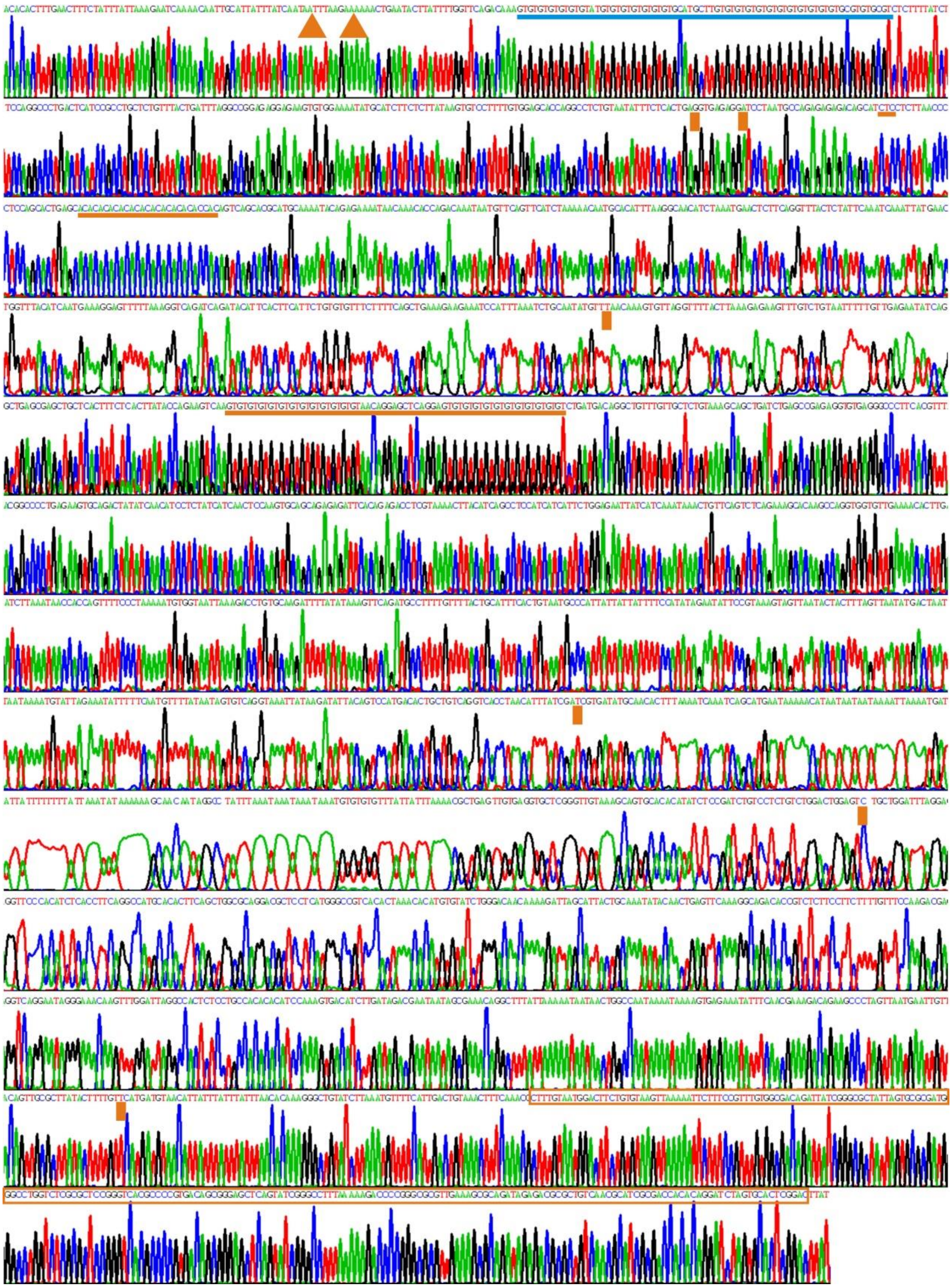

Figure S6. Sequencing spectrums of the recombinant Chinese GF *ntl* promoter in the gynogenetic heterozygous diploid individual 2 (GHD 2). Orange arrow heads indicate the sites of Chinese GF specific deletions. Orange and blue underlines indicate the Chinese GF and Japanese GF specific sequences, respectively. Orange and blue boxes indicate the Chinese GF and Japanese GF specific SNP sites, respectively. Open orange rectangle indicates the Chinese GF CpG island.
